# Hepatic Glycogenosis: A Rare Complication of Type 1 Diabetes Mellitus

**DOI:** 10.7759/cureus.74766

**Published:** 2024-11-29

**Authors:** Chaima Jemai, Yasmine Fakhfakh, Zohra Hadj Ali, Yosra Htira, Faika Ben Mami

**Affiliations:** 1 Department C, National Institute of Nutrition of Tunis, Tunis, TUN

**Keywords:** education, hepatic glycogenosis, hepatomegaly, mauriac syndrome, type 1 diabetes mellitus (t1dm)

## Abstract

Type 1 diabetes mellitus (T1DM) is a common autoimmune pathology requiring lifelong insulin therapy. We report the case of a 12-year-old girl with T1DM admitted to Department C of the National Institute of Nutrition of Tunis for diabetic ketosis. She had suffered from T1DM for five years, with poor glycemic control (hemoglobin A1C = 10%) and poor therapeutic adherence. On examination, she had abdominal bloating with homogeneous hepatomegaly. Her height was 146 cm (less than the second percentile), her weight was 38 kg (less than the second percentile), and her body mass index was 17.8 kg/m². Tanner’s stage was S1P1A1. Biological investigations showed mixed dyslipidemia, normal liver and renal functions, and normal thyroid-stimulating hormone levels. The aspartate aminotransferase/alanine aminotransferase ratio was 1.35. Ultrasound of the abdomen revealed hepatomegaly with a liver span of 19 cm. Based on the clinical history and investigations, Mauriac syndrome was the most likely diagnosis of our patient. A holistic multidisciplinary approach, in collaboration with the child psychiatrist, was opted to optimize diabetes management and reduce hepatic metabolic overload. Further investigations were conducted to rule out differential diagnoses, especially viral and autoimmune hepatitis. Poor acceptance of type 1 diabetes leads to non-compliance with insulin therapy. Then, energy metabolism becomes defective with growth retardation and pubertal delay. Glucose accumulates in the liver leading to metabolic liver disease. Liver damage could be irreversible. Therapeutic education, a good doctor-patient relationship, and family support are the cornerstones of managing T1DM diabetes complicated by Mauriac syndrome.

## Introduction

Type 1 diabetes mellitus (T1DM) is a chronic disease characterized by autoimmune destruction of pancreatic beta cells, which secrete insulin, a key hormone in glycemic homeostasis [1]. Patients with T1DM require life-long insulin replacement.

Novel technologies, such as automated insulin delivery systems, have substantially impacted the quality of life and the diabetes balance of these patients worldwide. However, these expensive technologies are unaffordable in developing countries such as Tunisia, where multiple daily insulin injections remain the only possible therapeutic option. Yet, this is very constraining in real life and could have many psychological issues and poor therapeutic compliance resulting in the development of related T1DM complications [2,3].

We aim to enrich the scientific literature by presenting this case. The case was previously presented as a meeting abstract at the 26th European Congress of Endocrinology.

## Case presentation

We report the case of a 12-year-old T1DM girl admitted to Department C of Diabetology and Therapeutic Dietetics of the National Institute of Nutrition of Tunis for diabetic ketosis. She had T1DM for five years with poor therapeutic compliance resulting in a hemoglobin A1c (HbA1c) level above the goal (HbA1c = 10%) and repeated emergency hospitalizations.

On physical examination, she had abdominal distension. The hepatic area was tender and the liver was enlarged. A familial history of hepatic disease was ruled out. She denied any symptoms such as fever, diarrhea, abdominal pain, nausea, and vomiting. She also denied medication, herbal intake, and viral infections. Her height, weight, and body mass index were 146 cm (less than the second percentile), 38 kg (less than the second percentile), and 17.8 kg/m², respectively. Tanner’s stage was S1P1A1. There were no other relevant findings.

Biological investigations showed mixed dyslipidemia and normal liver and renal functions. Viral serologies (hepatitis B and C, cytomegalovirus, and Epstein-Barr virus) and autoimmune panel (anti-nuclear, anti-LKM1, anti-smooth muscle, and anti-mitochondrial antibodies) were negative. Thus, the differential diagnoses of viral and autoimmune hepatitis were ruled out. Laboratory findings are detailed in Table 1.

**Table 1:** Laboratory findings of the patient. HbA1c: hemoglobin A1c; HDL: high-density lipoprotein; TSH: thyroid-stimulating hormone; AST: aspartate aminotransferase; ALT: alanine aminotransferase

	Patient’s value	Normal range
Fasting glycemia (mmol/L)	13.1	4.1–6.1
HbA1c (%)	10	4.5–6
Triglyceridemia (mmol/L)	3.13	0.4–1.7
Cholesterolemia (mmol/L)	6.14	3–5.2
HDL-cholesterol (mmol/L)	1.73	>1.03
Creatinemia (µmol/L)	20.4	135–145
TSH (IU/L)	3.07	0.34–5.6
AST (IU/L)	65	<45
ALT (IU/L)	48	<40
AST/ALT ratio	1.35	

Ultrasound examination revealed homogenous hepatomegaly with a liver span of 19 cm. Intrahepatic biliary ducts showed no ectasia, and the gallbladder had no calculi. Uncontrolled diabetes, hepatomegaly, growth retardation, impuberism, dyslipidemia, and the aspartate aminotransferase/alanine aminotransferase ratio >1 were features in favor of Mauriac syndrome in our patient.

## Discussion

Mauriac syndrome was first described in 1930. It is a rare complication of diabetes mellitus typically diagnosed in children and adolescents with poorly controlled T1DM [4]. In our case, many features were noted such as unbalanced diabetes, hepatomegaly, growth retardation, impuberism, cushingoid features, hypercholesterolemia, and elevated liver transaminases [5]. No presentation is specific to this syndrome, as one or more features could coexist in the same patient, but hepatomegaly and elevated liver are leading symptoms [6,7]. The full pathogenesis of Mauriac syndrome has not been fully elucidated. However, it is mainly characterized by glycogen accumulation in the hepatocytes.

In these patients, chronic hyperglycemia is often associated with high doses of insulin to try to control diabetes. Insulin inhibits glucose-6-phosphatase and stimulates glucokinase and glycogen synthetase which promotes hepatic glycogenogenesis. Thereby, chronic hyperglycemia and hyperinsulinism seem to act in combination to enhance hepatic glycogenogenesis with a remarkable snowball effect resulting in progressive enlargement of hepatocytes due to glycogen accumulation and clinical hepatomegaly [7-9].

Stature and puberty retardation are other common features of Mauriac syndrome in children. Their pathogenesis seems to be enhanced by glucogen liver sequestration associated with an insufficient supply of glucose to different tissues [10].

Some studies have reported a complete reversibility of symptoms through optimal glycemic control [11-14]. Thereby, a holistic multidisciplinary approach, in collaboration with the child psychiatrist, was opted in our case to optimize diabetes management and reduce hepatic metabolic overload, while achieving a resumption of height and weight growth and secondarily physiological puberty. Therapeutic education was resumed and insulin doses were adjusted according to glycemic cycles. Regular and timely consultations were planned with the patient and her family for further follow-up.

## Conclusions

Despite scientific progress, T1DM management continues to be challenging in some cases, such as those complicated with Mauriac syndrome. This is a rare complication noted in young patients with uncontrolled T1DM and chronic hyperglycemia resulting in hepatic accumulation of glycogen and leading to metabolic liver disease with possible irreversible liver damage. Subsequently, energy metabolism becomes defective with growth retardation and pubertal delay. Thus, caregivers should coordinate to enhance the care of patients with T1DM. Therapeutic education, a good doctor-patient relationship, and family support are the main cornerstones of managing T1DM complicated by Mauriac syndrome.
